# Supplementary material for: A Comprehensive Biomarker Analysis of Microsatellite Unstable/Mismatch Repair Deficient Colorectal Cancer Cohort Treated with Immunotherapy
Source: Int J Mol Sci. 2022 Dec 21;24(1):118. doi: 10.3390/ijms24010118 (PMC9820517; doi:10.3390/ijms24010118)
Supplement: Supplementary file 1 [file ijms-24-00118-s001.zip › ijms-2010270-supplementary.pdf]

Supplementary Table S1. This table summarizes: 1. Origin of samples used for study procedures 2. Anti-neoplastic treatments administered to patients before sample collection 3. Immune microenvironment analysis (regarding $CD3^+$, $CD8^+$, $FOXP3^+$ densities and PD-L1 expression) performed depending on tissue availability 3. IHC and PCR tests centrally performed for diagnosis of MSI-H/dMMR phenotype depending on tissue availability.

Chemo: chemotherapy. CPC: capecitabine. NE: non evaluable. NP: not performed. P: performed. Perit: peritoneal node. Prim: primary tumour. Pts: patients. RT: radiotherapy. *Patient 14 received FOLFOX plus panitumumab and FOLFIRI plus bevacizumab before sample collection.

Pts	Sample origin	Previous treatments	CD3	CD8	FOXP3	PD-L1	IHC	PCR
1	Prim	None	P	P	P	P	P	P
2	Prim	None	P	P	P	P	P	P
3	Prim	None	P	P	P	P	P	P
4	Prim	None	P	P	P	P	P	P
5	Prim	None	P	**NP**	P	P	P	P
6	Prim	**RT/ CPC**	**NP**	**NP**	**NP**	**NP**	P	NE
7	Prim	None	P	P	P	**NP**	P	P
8	Prim	None	**NP**	**NP**	**NP**	**NP**	**NP**	P
9	Prim	None	P	P	P	P	P	P
10	Prim	None	P	P	P	**NP**	P	P
11	**Perit**	None	**NP**	**NP**	**NP**	**NP**	P	P
12	Prim	None	P	P	P	P	P	P
13	Prim	None	P	P	P	P	P	P
14	Prim	**Chemo***	P	P	P	P	P	P
15	Prim	None	P	P	P	P	P	P
16	Prim	None	**NP**	**NP**	**NP**	**NP**	P	P

Supplementary Table S2. NGS panel designed for the analysis of FFPE-derived genomic DNA.

ABL1	BRAF	CSF1R	FAM123B	GATA6	IRS1	MDM2	NTRK3	PRKAR1A	RUNX1T1	TERC
ABL2	BRCA1	CTCF	FAM175A	GID4	IRS2	MDM4	NUP93	PRKCI	RYBP	TERT
ACVR1B	BRCA2	CTLA4	FAM46C	GLI1	JAK1	MED12	PAK1	PRKDC	SDHA	TET1
AKT1	BRD4	CTNNA1	FANCA	GNA11	JAK2	MEF2B	PAK3	PRSS8	SDHAF2	TET2
AKT2	BRIP1	CTNNB1	FANCC	GNA13	JAK3	MEN1	PAK7	PTCH1	SDHB	TGFBR1
AKT3	BTG1	CUL3	FANCD2	GNAQ	JUN	MET	PALB2	PTEN	SDHC	TGFBR2
ALK	BTK	CYLD	FANCE	GNAS	KAT6A	MITF	PARK2	PTPN11	SDHD	TMEM127
ALOX12B	C11orf30	DAXX	FANCF	GPR124	KDM5A	MLH1	PARP1	PTPRD	SETD2	TMPRSS2
AMER1	CARD11	DCUN1D1	FANCG	GREM1	KDM5C	MLL	PAX5	PTPRS	SF3B1	TNFAIP3
APC	CASP8	DDR2	FANCL	GRIN2A	KDM6A	MLL2	PBRM1	PTPRT	SH2D1A	TNFRSF14
AR	CBFB	DICER1	FANCM	GRM3	KDR	MLL3	PDCD1	QKI	SHH	TOP1
ARAF	CBL	DIS3	FAS	GSK3B	KEAP1	MPL	PDCD1LG2	RAC1	SHQ1	TOP2A
ARFRP1	CCND1	DNMT1	FAT1	H3F3A	KEL	MRE11A	PDGFRA	RAD50	SLIT2	TP53
ARID1A	CCND2	DNMT3A	FBXW7	H3F3C	KIT	MSH2	PDGFRB	RAD51	SMAD2	TP63
ARID1B	CCND3	DNMT3B	FGF10	HGF	KLF4	MSH6	PDK1	RAD51B	SMAD3	TRAF7
ARID2	CCNE1	DOT1L	FGF14	HIST1H1C	KLHL6	MTOR	PDPK1	RAD51C	SMAD4	TSC1
ARID5B	CD274	E2F3	FGF19	HIST1H2BD	KMT2A	MUTYH	PHOX2B	RAD51D	SMARCA4	TSC2
ASXL1	CD276	EED	FGF23	HIST1H3B	KMT2C	MYC	PIK3C2B	RAD52	SMARCB1	TSHR
ASXL2	CD79A	EGFL7	FGF3	HNF1A	KMT2D	MYCL	PIK3C2G	RAD54L	SMARCD1	U2AF1
ATM	CD79B	EGFR	FGF4	HRAS	KRAS	MYCL1	PIK3C3	RAF1	SMO	VEGFA
ATR	CDC73	EIF1AX	FGF6	HSD3B1	LATS1	MYCN	PIK3CA	RANBP2	SNCAIP	VHL
ATRX	CDH1	EP300	FGFR1	HSP90AA1	LATS2	MYD88	PIK3CB	RARA	SOCS1	VTCN1
AURKA	CDK12	EPCAM	FGFR2	ICOSLG	LMO1	MYOD1	PIK3CD	RASA1	SOX10	WISP3
AURKB	CDK4	EPHA3	FGFR3	IDH1	LRP1B	NBN	PIK3CG	RB1	SOX17	WT1
AXIN1	CDK6	EPHA5	FGFR4	IDH2	LYN	NCOR1	PIK3R1	RBM10	SOX2	XIAP
AXIN2	CDK8	EPHA7	FH	IFNGR1	LZTR1	NF1	PIK3R2	RECQL4	SOX9	XPO1
AXL	CDKN1A	EPHB1	FLCN	IGF1	MAD2L2 (REV7	NF2	PIK3R3	REL	SPEN	YAP1
B2M	CDKN1B	ERBB2	FLT1	IGF1R	MAGI2	NFE2L2	PIM1	RET	SPOP	YES1
BAP1	CDKN2A	ERBB3	FLT3	IGF2	MALT1	NFKBIA	PLCG2	RFWD2	SPTA1	ZBTB2
BARD1	CDKN2B	ERBB4	FLT4	IKBKE	MAP2K1	NKX2-1	PLK2	RHEB	SRC	ZNF217
BBC3	CDKN2C	ERCC2	FOXA1	IKZF1	MAP2K2	NKX3-1	PMAIP1	RHOA	STAG2	ZNF703
BCL2	CEBPA	ERCC3	FOXL2	IL10	MAP2K4	NOTCH1	PMS1	RICTOR	STAT3	
BCL2L1	CHD2	ERCC4	FOXP1	IL7R	MAP3K1	NOTCH2	PMS2	RIT1	STAT4	
BCL2L11	CHD4	ERCC5	FRS2	INHBA	MAP3K13	NOTCH3	PNRC1	RNF43	STK11	
BCL2L2	CHEK1	ERG	FUBP1	INPP4A	MAP3K5	NOTCH4	POLD1	ROS1	STK40	
BCL6	CHEK2	ERRFI1	GABRA6	INPP4B	MAPK1	NPM1	POLE	RPS6KA4	SUFU	
BCOR	CIC	ESR1	GATA1	INSR	MAPK7	NRAS	PPP2R1A	RPS6KB2	SUZ12	
BCORL1	CREBBP	ETV1	GATA2	IRAK4	MAX	NSD1	PPP2R2A	RPTOR	SYK	
BLM	CRKL	ETV6	GATA3	IRF2	MCL1	NTRK1	PRDM1	RSPO2	TAF1	
BMPR1A	CRLF2	EZH2	GATA4	IRF4	MDC1	NTRK2	PREX2	RUNX1	TBX3	

Supplementary Materials. Library preparation

DNA extraction from FFPE samples (ten 10-µm FFPE tissue sections) was performed with the automated system Maxwell16 FFPE plus LEV DNA purification kit (Promega). Briefly, 500 nanograms of purified DNA previously sheared into 150-200 base pairs fragments (Covaris S2), followed by library preparation according to manufacturer's instructions (Agilent SureSelect). The genes of interest were captured using the biotinylated custom baits from Agilent SureSelect customized oligo pool. All samples were sequenced on an Illumina platform to an average coverage of x415.

Supplementary Materials. Bioinformatic analysis

Libraries were sequenced in a HiSeq2500 instrument (Illumina), 2X100 Paired-end. Sequencing reads were quality controlled with FastQC and mapped to the human reference (hg19) with bwa (v.0.7.17) with default settings. The resulting BAM files were processed using SAMtools (v.1.7) and the Genome Analysis ToolKit (GATK) release 3.7. Variants were called using VarScan (v2.4.3), Mutect (v2) and Strelka with the following parameters: minimum variant allele frequency (VAF) of 5%, minimum coverage of 8 reads, at least 7 reads that confirm the mutation with a p-value below 0.05. Due to the hypermutant nature of the MSI-H/dMMR tumors, only mutations detected with at least two of the three variant callers were considered for further analysis.

Filtering of frequent SNPs in the population was accomplished with the 1000 genome database [1], the exome variant server database [2] and the GnomAD database [3] according to a population VAF > 0.0001 described in GnomAD [4]. Clinical significance classification of the variants was performed using the following databases COSMIC[5], cBioPortal[6], ClinVar[7], OncoKB[8] and VarSome[9].

Manual curation of the data was performed among all the knowledge databases for harmonization of their criteria.

For the calculation of copy number alterations (CNA), the tool CNVkit based on a parent –specific copy number segmentation method (PSCBS) was used; each tumour was compared with a home-made second reference sample created by pooling DNA from 150 tumours extracted from FFPE tissues from different tumour types, and mixed in equal amounts.

For the analysis of loss of heterozygosity (LOH) the SNP backbone captured data was used, the analysis was performed with SAMtools (v.1.7.) and results were plotted with ggplot2.

Supplementary Materials. <u>Immune microenvironment analysis</u>

IHC was performed on consecutive sections using CD3, CD8, FOXP3, PD-L1 and Pan-Keratin antibodies. Briefly, the slides were heated and deparaffinized before heat-induced antigen retrieval. Primary antibodies were applied as indicated in the following table.

**Antigen**	**Clone**	**Dilution**	**Manufacturer and reference**	**Primary antibody incubation time and temperature**
CD3	2GV6	Ready-to-use	Ventana Medical Systems (# 790-4341)	32 min 36ºC
CD8	144B	1/100	Dako Agilent (#M7103)	32 min RT
FOXP3	SP97	1/100	ABCAM	60 min RT
PD-L1	SP263	Ready-to-use	Ventana Medical Systems (#741-4905)	16 min 36ºC
Pan Keratin	AE1/AE3 & PCK26	Ready-to-use	Ventana Medical Systems (#760-2595)	36 min RT

Reactions were detected using the UltraView Universal DAB Detection kit. Finally, the slides were counterstained with Haematoxylin and mounted with a Xylol based mounting medium. The entire process was performed in the Benchmark ULTRA system and all reagents were from Ventana Medical Systems. Slides were digitalized using a slide scanner (NanoZoomer 2.0-HT, Hamamatsu Photonics, Japan) and quality checked by a pathologist before DIA. For IHC staining quantification, we used different image analysis algorithms created through the Author™ module of Visiopharm®. CD3, CD8 and PD-L1 slides were aligned with the Pan-Keratin slide using Tissuealign® (Visiopharm®). In the case of CD3, CD8 and FOXP3, Pan-Keratin was used to select the tumour and the surrounding stroma (200μm from the tumour) areas

where densities of stained immune cells were calculated. For PD-L1 analysis, the Composite Positive Score (CPS) was calculated dividing the number of PD-L1 positive cells by the total number of Pan-Keratin positive tumour cells multiplied by 100[10].

Supplementary material. **<u>Statistical analysis</u>**

A descriptive analysis of different variables included in the study was performed. Continuous variables were expressed as mean, median and interquartile range (IQR), and categorical variables were expressed as absolute values and percentages.

For univariate analysis, we used the Fisher's exact test for categorical variables and Student's *t* test or Mann-Whitney test for continuous variables, as appropriate after checking normality with the Shapiro-Wilk test. Spearman's correlation was used to evaluate association between continuous variables.

Survival analysis was calculated using the Kaplan-Meier method and log-rank test was used for statistical comparisons. Cox proportional-hazard models were used to obtain hazard ratios (HRs) with 95% confidence intervals (CIs). All P values were two-sided, and values less than 0.05 were considered statistically significant. In case of multiple testing, P values were adjusted using the Benjamini and Hochberg method.

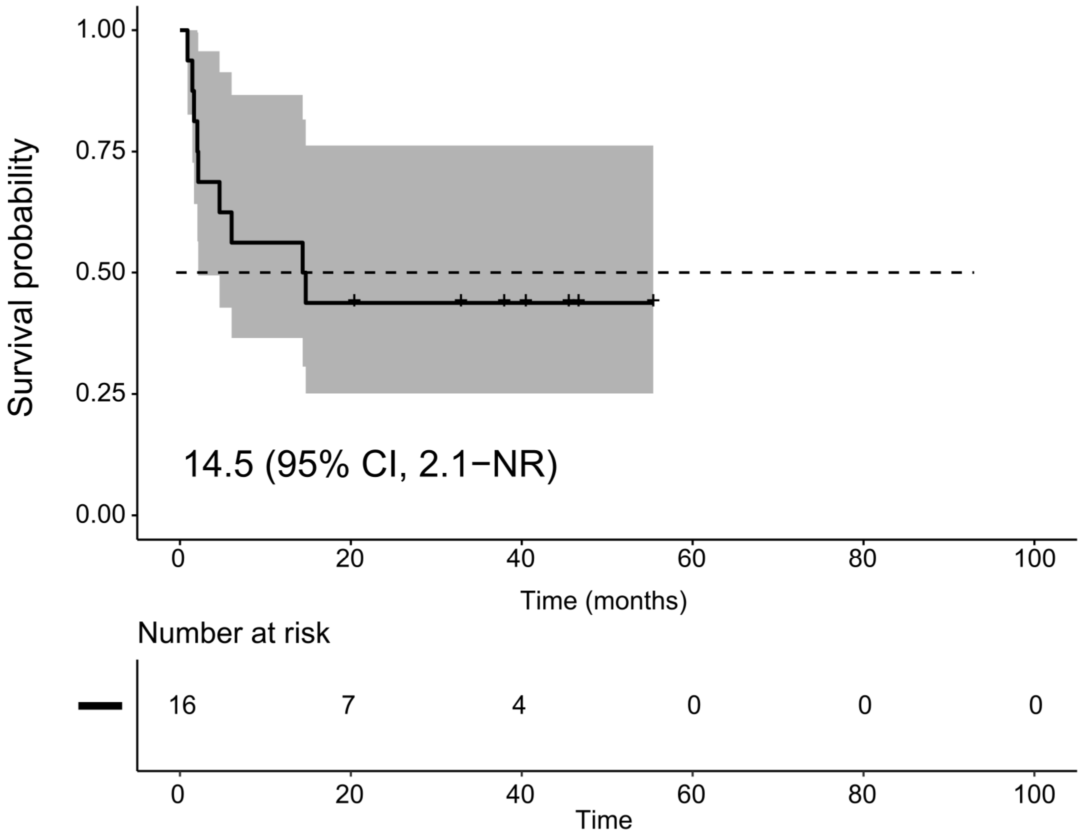

Supplementary Figure S1. Progression-free survival (PFS) curve and confidence intervals (grey area) for the entire cohort. The median PFS was 14.5 months. NR: not reached.

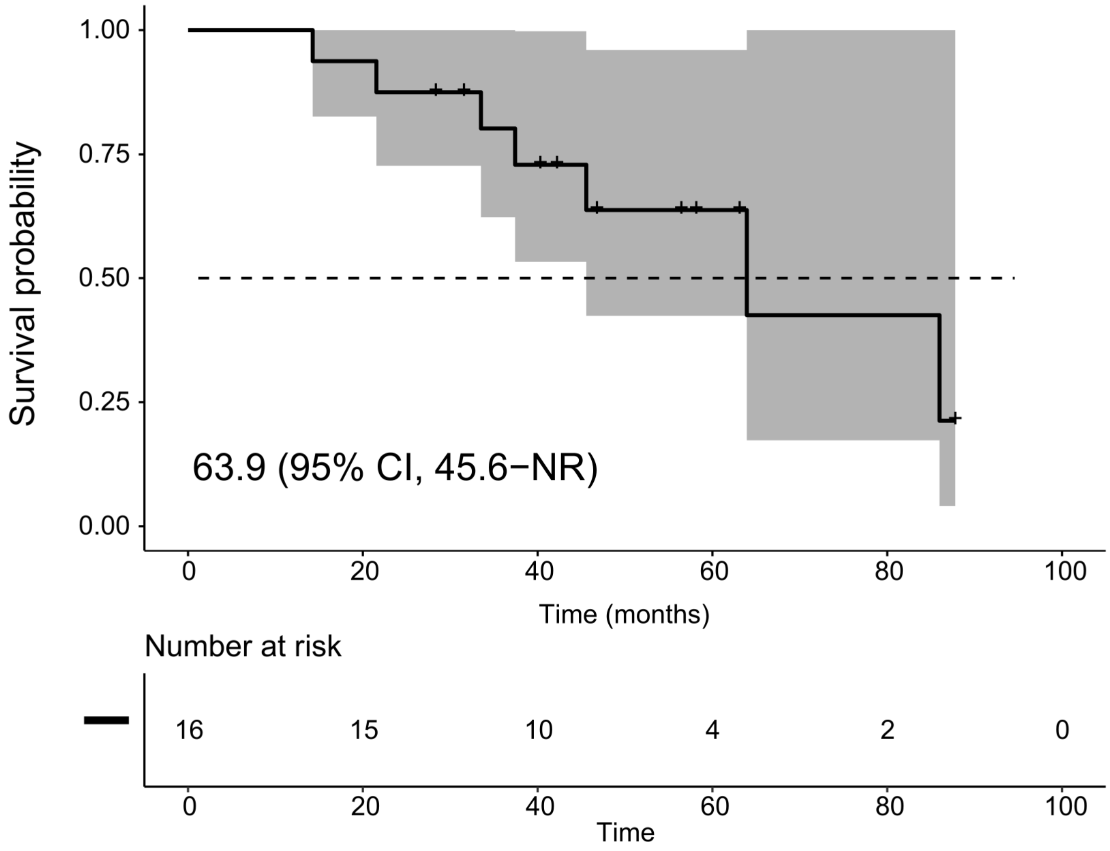

Supplementary Figure S2. Overall survival (OS) curve and confidence intervals (grey area) for the entire cohort. The median OS of metastatic disease was 63.9 months. NR: not reached.

Supplementary Excel S1. – (*file "NGS results MSI final*). Results of NGS panel, CNA and LOH for each patient included in the study. Analysis of LOH and CNA provided homogeneous results for all patients, reporting no alterations in any of the samples, except for a single copy loss of *NF1* gene in patient 6.

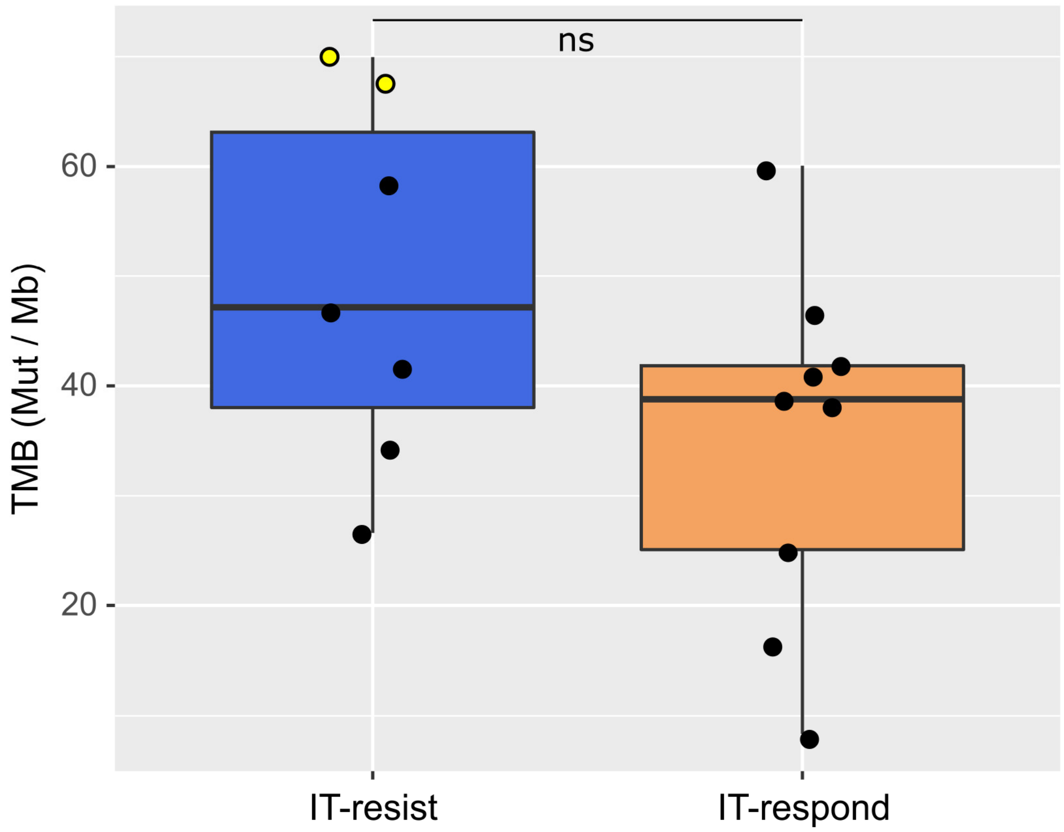

Supplementary Figure S3. TMB (mutations/Mb) was calculated for each patient (dots) according to mutations detected in the NGS targeted panel. Median value of TMB is: 47.2 mut/Mb in IT-resistant, and 38.8 mut/Mb in IT-responder. The differences are not statistically significant. Yellow dots represent patients with the highest TMB. ns: not significant.

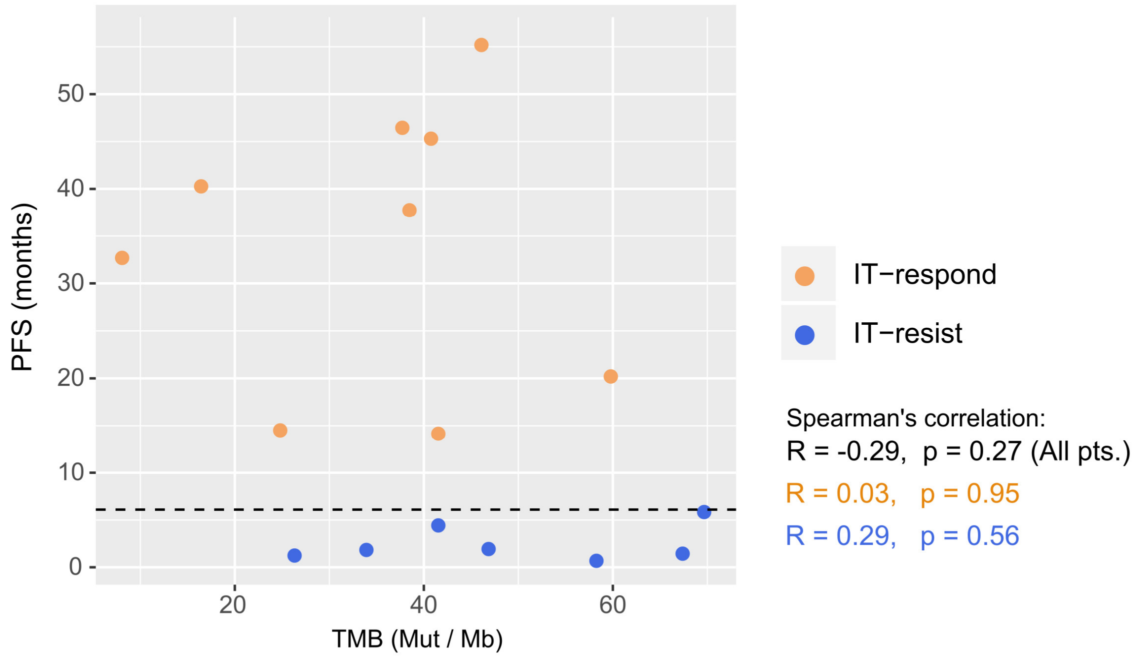

Supplementary Figure S4. Correlation between PFS (months) and TMB (mut/Mb). A trend is observed suggesting higher TMB correlates with worse PFS, but without statistical significance. Dashed line indicated the previous defined cut-off of 6 months for PFS. pts: patients.

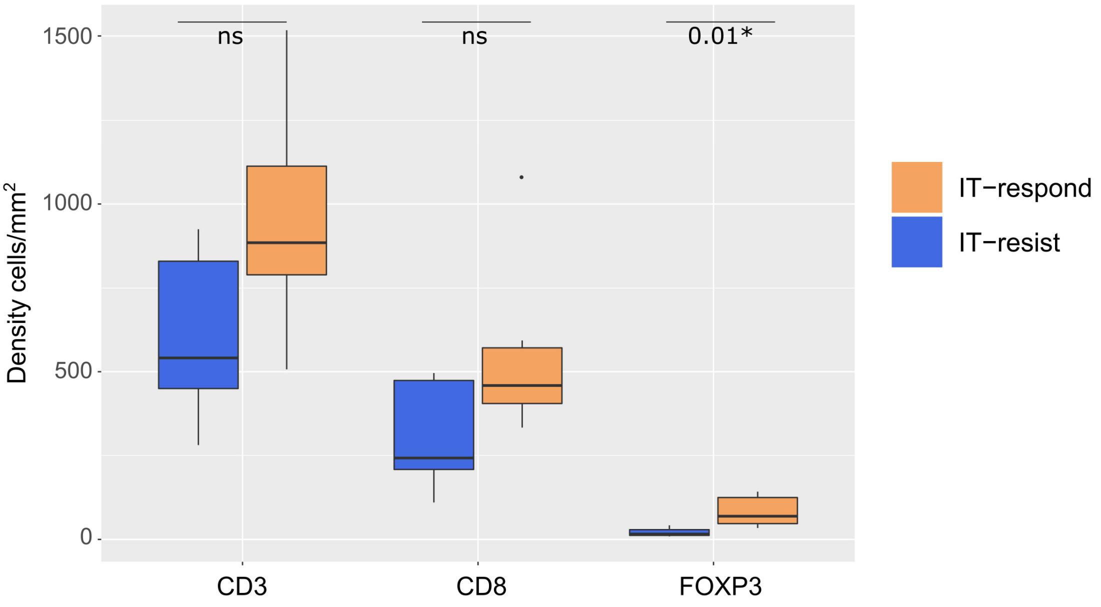

Supplementary Figure S5. Densities of $CD3^+$, $CD8^+$ and $FOXP3^+$ lymphocytes according to IT-responder and IT-resistant. Median values are higher in IT-responder, reaching the statistical significance in $FOXP3^+$ subpopulation. ns: not significant.

Supplementary Excel S2. Excel detailing data related with Figure 3.

	IT-respond									IT-resist						
**Patient**	**1**	**2**	**3**	**4**	**5**	**6**	**7**	**8**	**9**	**10**	**11**	**12**	**13**	**14**	**15**	**16**
**Mechanism of dMMR/MSI-H phenotype**	Germline	Germline	Germline	Sporadic	Sporadic	Sporadic	Germline	Sporadic	Sporadic	Sporadic	Sporadic	Sporadic	Sporadic	Sporadic	Sporadic	Sporadic
APC	wt	wt	wt	wt	wt	mut	wt	wt	wt	mut	wt	wt	wt	mut	wt	wt
APC Bi	mut	mut	mut	wt	wt	wt	mut	wt	wt	wt	wt	wt	mut	wt	wt	wt
TP53	wt	wt	wt	wt	wt	mut	wt	wt	wt	wt	wt	mut	wt	wt	wt	wt
KRAS	mut	mut	wt	wt	wt	wt	mut	wt	wt	mut	wt	wt	mut	wt	wt	wt
BRAF V600E	wt	wt	wt	mut	mut	wt	wt	mut	wt	wt	wt	mut	wt	mut	mut	wt
EGFR	wt	wt	wt	wt	wt	wt	wt	wt	wt	mut	mut	wt	wt	wt	wt	wt
PIK3CA	mut	mut	wt	wt	wt	wt	wt	wt	mut	wt	wt	wt	wt	mut	wt	wt
RNF43	wt	wt	wt	wt	mut	wt	wt	mut	mut	wt	mut	mut	wt	wt	wt	wt
CTNNB1	wt	wt	wt	wt	wt	wt	wt	wt	wt	mut	wt	wt	wt	wt	wt	mut
B2M	wt	wt	mut	wt	wt	wt	wt	wt	mut	wt	wt	mut	wt	mut	mut	wt
B2M Bi	wt	wt	wt	wt	wt	wt	wt	wt	wt	wt	mut	wt	wt	wt	wt	wt
JAK 1,2,3	wt	wt	mut	wt	wt	wt	mut	wt	wt	wt	wt	mut	wt	mut	wt	wt
PTEN	wt	wt	wt	wt	wt	wt	wt	wt	mut	wt	wt	wt	wt	wt	wt	wt
PTEN Bi	wt	wt	wt	mut	wt	wt	wt	wt	wt	mut	wt	mut	wt	wt	wt	mut
ARID1A	wt	wt	wt	mut	wt	wt	wt	wt	wt	wt	wt	wt	mut	wt	mut	mut
ARID1A Bi	wt	mut	wt	wt	mut	wt	mut	wt	mut	wt	wt	wt	wt	wt	wt	wt
MLH1	wt	mut	wt	wt	wt	wt	mut	wt	mut	wt	wt	wt	mut	wt	wt	mut
MLH1 Bi	wt	wt	mut	wt	wt	wt	wt	wt	wt	wt	wt	wt	wt	wt	wt	wt
MSH2	wt	wt	wt	wt	wt	wt	wt	wt	wt	wt	wt	wt	wt	wt	wt	wt
MSH2 Bi	mut	wt	wt	wt	wt	wt	wt	wt	wt	wt	wt	wt	wt	wt	wt	wt
MSH6	mut	wt	wt	wt	wt	wt	wt	mut	wt	wt	mut	wt	wt	mut	wt	mut
MSH6 Bi	wt	wt	wt	wt	wt	wt	mut	wt	wt	wt	wt	wt	wt	wt	wt	wt
**TMB (Mut/Mb)**	46,39	38,02	41,06	16,73	38,78	8,37	60,08	25,10	41,83	69,96	41,83	47,15	34,22	67,68	26,62	58,56
**fs (indels)**	9,0	11,0	15,0	4,0	20,0	2,0	8,0	13,0	16,0	24,0	14,0	18,0	11,0	16,0	7,0	24,0
**% fs vs TOTAL**	14,8	22,0	27,8	18,2	39,2	18,2	10,1	39,4	29,1	26,1	25,5	29,0	24,4	18,0	20,0	31,2
**COSMIC signature Defective DNA MMR / MSI (small INDELs)**	0,7	0,4	0,3	0,7	0,6	0,5	0,3	0,2	0,5	0,6	0,9	0,3	0,4	0,5	0,5	0,3
**COSMIC signature Others**	0,3	0,6	0,7	0,3	0,4	0,5	0,7	0,8	0,5	0,4	0,1	0,7	0,6	0,5	0,5	0,7
**DENSITY CD8/mm²**	334,0	1081,0	505,0	403,0	NA	NA	593,0	NA	413,0	110,0	NA	242,0	496,0	208,0	474,0	NA
**DENSITY CD3 /mm²**	507,0	1517,0	885,0	884,0	695,0	NA	1170,0	NA	1055,0	282,0	NA	450,0	830,0	541,0	925,0	NA
**DENSITY FOXP3/mm²**	40,0	140,0	109,0	54,0	69,0	NA	142,0	NA	34,0	9,0	NA	29,0	42,0	12,0	16,0	NA
**PD-L1 CPS**	1,0	30,0	5,0	10,0	90,0	NA	NA	NA	0,5	NA	NA	5,0	5,0	0,5	2,0	NA
**IHC results (prot expression lost)**	MSH2	MLH1/PMS2	MLH1/PMS2	MLH1/PMS2	MLH1/PMS2	MLH1	MSH6	NA	MLH1/PMS2	MLH1/PMS2	MLH1/PMS2	MLH1/PMS2	MLH1/PMS2	PMS2	MLH1/PMS2	MLH1/PMS2
**Number of unstable microsatellites (PCR)**	5	4	5	5	5	NA	3	4	4	0	3	1	4	3	0	4
**PROLIFERATING CD3**	9	16	36	3	13	NA	28	NA	8	1	NA	14	9	7	6	NA
**PROLIFERATING CD8**	12	20	NA	5	NA	NA	35	NA	11	2	NA	14	17	12	NA	NA
**PROLIFERATING FOXP3**	8	24	27	5	16	NA	33	NA	12	0	NA	17	15	13	15	NA
***Fusobacterium nucleatum* (bacteria/mm²)**	0,9	246,3	NA	3,7	63,2	NA	0,1	NA	0,6	466,7	0	10,2	5	8,2	0,2	NA

SUPPLEMENTARY MATERIAL REFERENCES

1. Altshuler DM, Durbin RM, Abecasis GR et al. An integrated map of genetic variation from 1,092 human genomes. Nature 2012; 491(7422):56–65.

2. esp.gs.washington.edu/drupal.

3. Lek M, Karczewski KJ, Minikel E V, et al. Analysis of protein-coding genetic variation in 60,706 humans. Nature 2016; 536(7616):285–291.

4. Karczewski KJ, Francioli LC, Tiao G, et al. The mutational constraint spectrum quantified from variation in 141,456 humans. Nature 2020; 581(7809):434–443.

5. COSMIC. Catalogue Of Somatic Mutations In Cancer. Available at: https://cancer.sanger.ac.uk. Year 2021.

6. cBioPortal for cancer genomics. Available at: https://cbioportal.org. Year 2021

7. ClinVar -NCBI-NIH. Available at: https://ncbi.nlm.nih.gov/clinvar/. Year 2021.

8. OncoKB. Available at: https://oncokb.org/. Year 2021

9. The Human Genomic Variant. Available at: https://varsome.com. Year 2021.

10. Kulangara K, Zhang N, Corigliano E, et al. Clinical utility of the combined positive score for programmed death ligand-1 expression and the approval of pembrolizumab for treatment of gastric cancer. Arch. Pathol. Lab. Med. 2019; 143(3):330–337.
